# Myopathy With Exercise‐Induced Intolerance due to Novel Biallelic Variants in *OBSCN*—A Clinical, Morphological and Molecular Analysis

**DOI:** 10.1111/nan.70065

**Published:** 2026-02-05

**Authors:** Heidrun H. Krämer‐Best, Marlen C. Reis, Andreas Hentschel, Michaela Weiß, Alexander Schaiter, Klaus‐Dieter Böhm, Andreas Roos, Dagmar Nolte, Anne Schänzer

**Affiliations:** ^1^ Department of Neurology Justus‐Liebig‐University Giessen Giessen Germany; ^2^ Translational Neuroscience Network Giessen (TNNG) Justus‐Liebig‐University Giessen Giessen Germany; ^3^ Institute of Human Genetics Justus‐Liebig‐University Giessen Giessen Germany; ^4^ Leibnitz‐Institut für Analytische Wissenschaften—ISAS—e.V. Dortmund Germany; ^5^ Institute of Neuropathology Justus‐Liebig‐University Giessen Giessen Germany; ^6^ BDH Klinik Braunfels Braunfels Germany; ^7^ Department of Pediatric Neurology, Centre for Neuromuscular Disorders University Duisburg‐Essen Essen Germany; ^8^ Brain and Mind Research Institute, Children's Hospital of Eastern Ontario Research Institute Ottawa Ontario Canada; ^9^ Department of Neurology, Medical Faculty and University Hospital Düsseldorf Heinrich Heine University Düsseldorf Germany

**Keywords:** autophagy, Ca^2+^ regulation, exercise intolerance, exercise‐induced myopathy, extrasarcomeric cytoskeleton, obscurin, sarcomere dysfunction, skeletal muscle

## Abstract

- The phenotype of *OBSCN* variants consists of exercise intolerance ranging from myalgia and cramps to rhabdomyolysis. The symptoms are mainly induced by high‐intensity sports.
- Two previously undescribed *OBSCN* variants have been identified as being associated with exercise intolerance, myotonic discharges and core‐like lesions in the muscle biopsy.
- Proteomic analysis of skeletal muscle reveals that the pathogenicity of the *OBSCN* variants is associated with dysregulated proteins that control Ca^2+^ handling and the extrasarcomeric cytoskeleton.

## Graphical abstract

The phenotype of *OBSCN* variants consists of exercise intolerance ranging from myalgia and cramps to rhabdomyolysis. Symptoms are mainly induced by high‐intensity sports. Molecular analysis showing a deregulation of muscle processes associated with Ca^2+^ regulation, extrasarcolemmal integrity and autophagy emphasised the critical role of obscurin in skeletal muscle function.

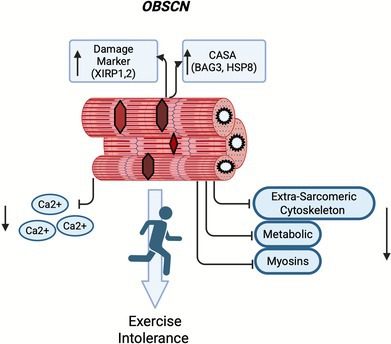

**Abbreviations**
CASA: chaperone‐assisted selective autophagy
DAP: differentially abundant protein
GO: gene ontology
IF: immunofluorescence
MS: mass spectrometry
SR: sarcoplasmic reticulum
TEM: transmission electron microscopy
VUS: variant of uncertain significance
WES: whole exome sequencing

Biallelic pathogenic variants in *OBSCN* (OMIM *608616) have been identified in nine patients aged 12–31 years who experienced exercise intolerance. In the majority of these patients, cramps, myalgia and rhabdomyolysis were triggered by high‐intensity sports [1, 2, 3].

*OBSCN* encodes obscurin, a giant structural protein of 720 kDa (obscurin‐A) or 870 kDa (obscurin‐B), which is primarily expressed in cardiac and skeletal muscle. Obscurin is a major component of sarcomeres interacting with titin, myomesin and small ankyrin 1 to form a ternary complex at sarcomeric M‐bands [4]. It acts as a linker between the sarcomere and the sarcoplasmic reticulum (SR) and is involved in calcium regulation [5, 6].

We present a 22‐year‐old woman who experienced muscle stiffness, myalgia and cramps in her thighs and gluteal muscles after performing high‐intensity sports (roundnet). Her first symptoms occurred at the age of 16 years. The proximal legs were predominantly affected. Additionally, hand muscles were involved. The symptoms appeared after exercise and gradually decreased over the next few days. Neurological examination revealed poor relaxation of handgrip following fist closure. Nerve conduction studies were normal. Electromyography revealed myotonic discharges in the vastus lateralis muscles, but no neurogenic changes were observed. Skeletal muscle MRI of the lower limbs showed hypertrophic proximal muscles without any other pathology (Figure 1A). Serum creatine kinase (CK) levels were mildly elevated (348 U/L; normal: 12–140 U/L). A cardiac workup was unremarkable. The patient's parents (II‐1 and II‐2) and her older twin siblings (III‐1 and III‐2) were healthy. The patient's maternal aunt (II‐3) had congenital muscle weakness and died from acute heart failure at the age of 12 (Figure 1B).

**FIGURE 1:**
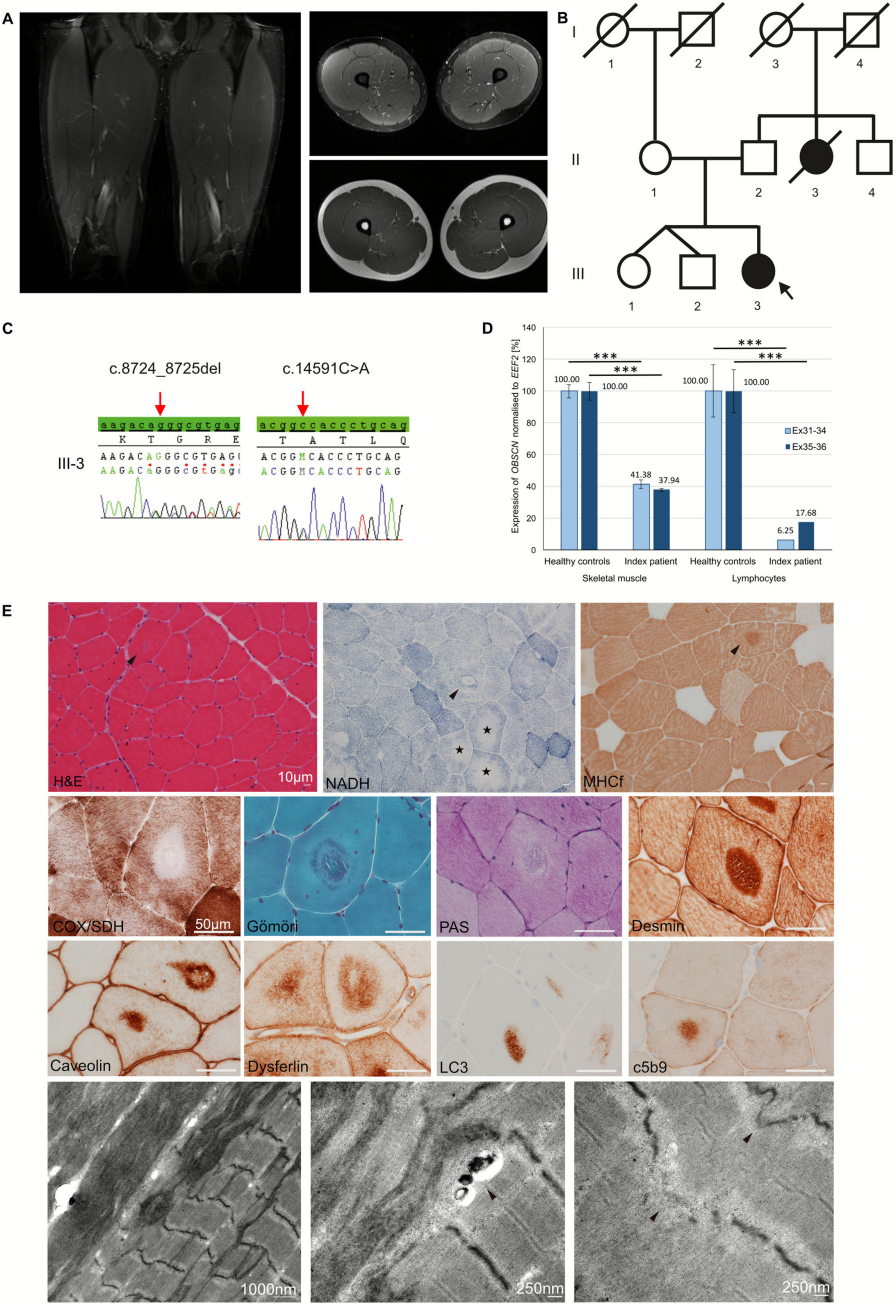
Skeletal muscle MRI (MR sequences: axial and coronal STIR and axial T1 FSE) of the proximal thighs of the patient showing hypertrophic muscles without oedema or fatty replacement (A). Pedigree of the family. Black symbols indicate affected probands. An arrow marks the index patient. A slash indicates deceased persons (B). Electropherograms of *OBSCN* sequences of the patient (C). *OBSCN* transcript expression was detected with two different primer pairs (covering exons 31 to 34 and exons 35 to 36) in skeletal muscle and lymphocytes. Fold change in *OBSCN* expression of the patient shows significantly decreased *OBSCN* expression compared to controls in skeletal muscle and lymphocytes; ***p < 0.001 (D). Muscle biopsy shows muscle fibres with a moderate increased variation in muscle fibre size, internalised nuclei (H&E) and predominance of type 2 fibres (MHCfast). Numerous fibres reveal central defects (arrows) with a surrounding dark rim (NADH, Gömöri, PAS) and moth‐eaten appearance in NADH (stars). Protein aggregates in the central lesions express desmin, caveolin‐3, dysferlin, autophagic marker LC3 and c5b9. Transmission electron microscopy (TEM) shows disruption of the myofibrillar architecture (arrow) with the presence of osmophilic material and small autophagosomes (arrow) (E).

Panel‐based genetic testing of the patient revealed six heterozygous variants of uncertain significance (VUS) and a heterozygous pathogenic frameshift variant in *CAPN3* (Table S1). Since these variants were present in the asymptomatic parents, they did not explain the symptoms.

Whole exome sequencing (WES) of a trio revealed a heterozygous frameshift variant in exon 33 (NM_001271223.3:c.8724_8725del, p.Gly2909Alafs*2) in *OBSCN*, leading to a premature stop codon in addition to a missense variant in exon 55 (NM_001271223.3:c.14591C > A, p.Ala4864Asp; ENST00000570156.7; Ensembl release 114) in the patient. The frameshift variant originated from the mother, while the father was a carrier of the *OBSCN* missense variant, as confirmed by Sanger sequencing (Figure 1C). Neither variant was reported in ClinVar nor in the 1000 Genomes database. According to ACMG guidelines [7], the frameshift variant p.Gly2909Alafs*2 was classified as pathogenic. In silico studies predicted a nonsense‐mediated decay for the *OBSCN* frameshift variant, most likely based on a premature stop codon at position 2911. To confirm this, quantitative PCR (qPCR) was performed on cDNA from skeletal muscle and lymphoblasts derived from the patient and healthy controls (two amplicons: primers for exon 31–34 and exon 35–36) (Table S2). *OBSCN* transcript levels were significantly decreased in the patient's muscle: 58.62% reduction for amplicon 1 (ex31–34), and a 62.06% reduction for amplicon 2 (ex35–36), and lymphoblasts: reductions of 93.75% (ex31–34) and 82.32% (ex35–36) (Figure 1D).

In silico predictions using PolyPhen2 and MutationTaster2 classified the missense variant p.Ala4864Asp as probably damaging or disease‐causing. The affected amino acid residue in obscurin is highly conserved. However, we classified the variant as a class III variant (VUS). Currently, there is limited data on *OBSCN* missense variants. These variants have been identified in patients with dilated cardiomyopathy (DCM). Several groups have studied some of these variants, such as p.Arg4344Gln and p.Arg4444Trp, but the pathogenic effects could not always be reproduced [8, 9]. The variant p.Ala4864Asp, described here for the first time, is in close proximity to p.Arg4856His, which was identified in a patient with DCM [10]. Although no heart involvement was detected in the patient, both variants might affect the same region of obscurin.

The patient's vastus lateralis muscle biopsy revealed a moderate increase in variability of fibre size, increased internalised nuclei (H&E) and a type 2 fibre predominance (MHCfast). Numerous fibres showed central defects with a surrounding dark rim and pale moth‐eaten appearance (NADH, Gömöri, PAS). Protein aggregates in the central lesions expressed desmin, caveolin3, dysferlin, c5b9 and autophagic marker LC3. Transmission electron microscopy (TEM) showed osmiophilic fil material with numerous small autophagosomes at the border and disruption of the myofibrillar structure with disruption of the M‐ and Z‐bands (Figure 1E).

With immunofluorescent staining (IF) using antibodies against desmin and obscurin, desmin expression was observed surrounding the sarcomeres and at the Z‐line in a control sample, whereas obscurin was expressed in alternating transverse striations at the M‐band. At the sarcolemma (cross sections), strong desmin expression and weak obscurin expression were seen. In the patient's muscle biopsy, desmin was expressed in the central lesions, while obscurin showed expression in a rim. Obscurin expression was irregular and reduced (Figure 2A). Details of antibodies are provided in Table S3.

**FIGURE 2:**
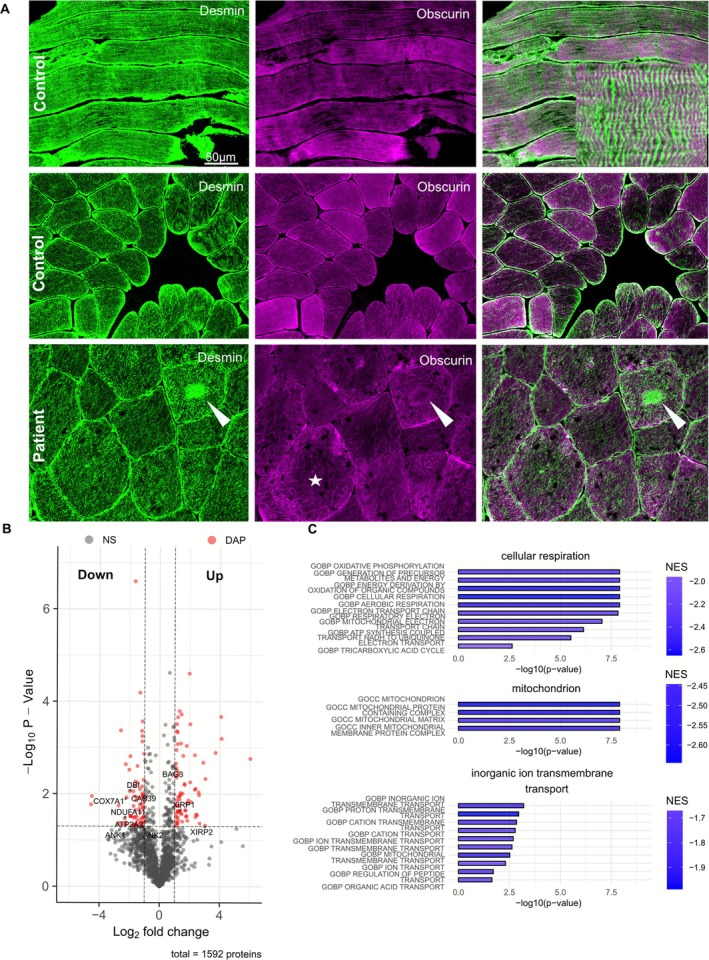
Double immunofluorescent analysis from a control shows alternating striatal expression of desmin and obscurin at longitudinal muscle section and a stronger sarcolemmal expression of desmin compared to obscurin in cross sections. In the patient, central lesions consist of desmin aggregates with a surrounding rim of obscurin expression (arrows). Overall, the expression of obscurin is more irregular and reduced in the patient's muscle fibres (star) (B). The Volcano plot shows 69 significantly downregulated and 70 upregulated abundant proteins (DAPs) (red dots). (C) Selected gene ontology (GO) terms reveal the downregulation of pathways related to cellular respiration, mitochondrial structure and ion transmembrane transport.

To better understand the molecular interplay between obscurin and other proteins, and the biological relevance of the loss of obscurin in striated muscle function, we performed proteomic profiling on muscle protein extracts derived from the index and three control muscle samples, as previously described [11]. Mass spectrometry (MS) revealed 69 significantly downregulated and 70 upregulated proteins (Figure 2B, Table S4). Increased proteins were associated with chaperone‐assisted selective autophagy (CASA) processes, including BAG family molecular chaperone regulator 3 (BAG3), heat shock protein beta‐8 (HSPB8) and muscle‐specific damage markers Xin actin‐binding repeat‐containing proteins 1 and 2 (XIRP1 and XIRP2) [12, 13]. In contrast, the decreased proteins impact extrasarcomeric cytoskeleton processes (ANK1 and ANK2) and Ca^2+^‐related and metabolic processes, as reported by other groups (Figure 2C) [5]. A reduced abundance of obscurin in muscle protein extracts derived from the patient compared to the control group was observed. Based on one outlier, our findings did not reach statistical significance (Figure S1). Biological processes associated with T‐tubule organisation, muscle contraction and calcium release into the cytosol by SR were downregulated (Figure S2). Gene functions and gene ontology (GO) terms revealed downregulation of processes related to cellular respiration, the mitochondria and inorganic ion transmembrane transport (Figure 2C).

Patients with biallelic variants of *OBSCN* exhibit exercise intolerance symptoms. Interestingly, the majority of the patients reported high‐intensity sports as a trigger [1, 2, 3]. Consistent with these findings, mice with obscurin deficiency exhibit exercise‐induced myofibrillar alterations that worsen with stronger exercise and older age [14].

The patient reported severe myalgia and cramps after high‐intensity sports, displaying poor relaxation of handgrip and myotonic discharges. Prolonged muscle contraction might result in pain, stiffness and sometimes cramping. Myotonic discharges seen on electromyography are typically caused by disorders affecting muscle membrane excitability [15]. Even though there was no rhabdomyolysis present in the patient at the current age (and at the last time the patient was seen in clinic), the data highlight that the two novel variants in *OBSCN* are most likely to be responsible for the patient's symptoms. Patients harbouring biallelic *OBSCN* nonsense variants can also present with exercise‐induced cramps and myalgia, as observed in our patient without developing the full clinical picture of rhabdomyolysis [1].

Previous studies of *OBSCN*‐associated myopathy revealed variable muscle pathology, including increased variation in muscle fibre size, internalised nuclei, ring fibres, necrotic fibres, predominance of type 2 muscle fibres and core‐like pathology [1, 2, 3].

The patient revealed a striking muscle pathology, including myofibrillar disintegration and aggregation of sarcomere proteins. The importance of obscurin in maintaining sarcomere integrity and stability during exercise is highlighted by the reduced expression of obscurin in the muscle fibre and increased expression as a surrounding rim around the central lesions [14]. Similar core‐like lesions have been described previously in a 39‐year‐old patient with biallelic loss of *OBSCN*. Interestingly, a sibling with the same biallelic *OBSCN* variants has not presented with rhabdomyolysis. This indicates the multifactorial nature of rhabdomyolysis, where an underlying genetic susceptibility combined with environmental triggers is often required for an individual to develop the condition [1].

We observed a deregulation of proteins affecting different biological functions, highlighting the overall relevance of the loss of functional obscurin for striated muscle function. Proteins associated with obscurin function, including the role in integrity of extrasarcomeric cytoskeleton, as a linker between sarcomere and SR and Ca^2+^‐related processes and its metabolic function, were downregulated [5]. This confirms the pathogenicity of the two variants identified in our patient. This also accords with findings that aberrant cytosolic Ca^2+^ flux is a hallmark of cell death in rhabdomyolysis and is disturbed in *OBSCN* patients [1]. In addition, the SR modulates cytosolic Ca^2+^ during contraction and relaxation, and disturbance of Ca^2+^ regulation is consistent with poor relaxation of handgrip following fist closure seen in the patient [16, 17]. Muscle pathology with sarcomeric aggregates was associated with upregulation of damage markers XIRP1, XIRP2 and CASA, BAG3 and HSPB8 [12, 13, 18]. These findings highlight common muscle processes in myopathies due to variants in sarcomeric proteins.

In summary, we present the clinical, morphological and molecular phenotypes of an *OBSCN*‐associated myopathy in an adolescent patient caused by novel biallelic *OBSCN* variants. The patient exhibited exercise‐induced symptoms and central myofibrillar disintegration in skeletal muscle fibres associated with sarcomeric aggregates. Molecular analysis revealed the downregulation of muscle processes associated with Ca^2+^ regulation and extrasarcolemmal integrity. This underscores the critical role of obscurin in skeletal muscle function.

## Author Contributions

A.S., D.N. and H.H.K.‐B. designed the study, analysed data and drafted the manuscript. M.C.R., M.W., Al.She. and A.He. conducted experimental research. A.R. performed data analysis and discussed data. H.H.K.‐B. provided clinical data. All authors have read and approved the final manuscript.

## Funding

A.S. and A.R. received funding from the Deutsche Gesellschaft für Muskelkranke (DGM e.V.). A.R. received funding from the European Regional Development Fund (ERDF; project NME‐GPS). A.H. acknowledges the support by the “Ministerium für Kultur und Wissenschaft des Landes Nordrein‐Westfalen” and “Der Regierende Bürgermeister von Berlin, Senatskanzlei Wissenschaft und Forschung.”

## Ethics Statement

Written informed consent was obtained from both the patient and her parents. This project was approved by the local ethics committee (AZ 07/09 Justus‐Liebig‐University Giessen).

## Conflicts of Interest

The authors declare no conflicts of interest.

## Supporting information


**Table S1:** Genetic findings.
**Table S2:** Primer sequences for (A) *OBSCN* sequencing and (B) quantitative RT‐PCR.
**Table S3:** Antibodies.
**Table S4:** Differentially abundant proteins (DAPs) in the index patient.

## Data Availability

Access to the proteomic data will be available upon reasonable request to the corresponding author.
