# Supplementary material for: Myopathy With Exercise‐Induced Intolerance due to Novel Biallelic Variants in OBSCN—A Clinical, Morphological and Molecular Analysis
Source: Neuropathol Appl Neurobiol. 2026 Feb 5;52(1):e70065. doi: 10.1111/nan.70065 (PMC12877425; doi:10.1111/nan.70065)
Supplement: Supplementary file 1 — Table S1: Genetic findings. Table S2: Primer sequences for (A) OBSCN sequencing and (B) quantitative RT‐PCR. Table S3: Antibodies. Table S4: Differentially abundant proteins (DAPs) in the index patient. [file NAN-52-e70065-s001.pdf]

Supplementary Material

Myopathy with exercise-induced intolerance due to novel biallelic variants in *OBSCN* – a clinical, morphological and molecular analysis

Heidrun H. Krämer-Best[1,2], Marlen C. Reis[3], Andreas Hentschel[4], Michaela Weiß[3], Alexander Schaiter[5], Klaus-Dieter Böhm[6], Andreas Roos[7,8,9], Dagmar Nolte[3]#, Anne Schänzer[5,2]#*

*Correspondence author: Anne Schänzer, anne.schaenzer@patho.med.uni-giessen.de; phone +49 641 99 41184; fax +49 641 99 41189

Material and Methods

Genetic analysis

The SureSelect XT Human HS Human All Exon V8 kit (Agilent Technologies, Santa Clara, CA, United States) was used for enrichment. Quality control of the prepared library was performed using a Qubit 3.0 fluorometer (ThermoFisher Scientific, Waltham, MA, United States). Sequencing was run on an Illumina NovaSeq platform (Illumina, San Diego, CA, United States). In all three DNA-samples, the mean coverage of exons was at least >20x with >99.4% targeted bases covered.

Filter criteria for pathogenic variants were a minor allele frequency (MAF) <0.01 in the gnomAD v2.1.1 database (Karczewski et al., 2020), and impact. The reference genome was GRCh37. Varvis software v.1.24.1 (Limbus Medical Technologies GmbH, Rostock, Germany) was used for analysis of single nucleotide variants (SNVs) and copy number variants (CNVs). Sanger sequencing was performed to reevaluate pathogenic and likely pathogenic variants.

Bioinformatic prediction

The impact of the observed variants was analyzed by *in silico* analysis using the programs MutationTaster2 (Schwarz et al., 2014), and PolyPhen2 (Adzhubei et al., 2010). The detected variants were subsequently classified according to the American College of Medical Genetics (ACMG) guidelines (Richards et al., 2015).

Adzhubei IA, Schmidt S, Peshkin L, Ramensky VE, Gerasimova A, Bork P, Kondrashov AS, Sunyaev SR (2010). Nat Methods 7:248-249. https://doi: 10.1038/nmeth0410-248

Schwarz J, Cooper D, Schuelke M, Seelow D (2014) MutationTaster2: mutation prediction for the deep-sequencing age. Nat Methods 11:361–362. https://doi.org/10.1038/nmeth.2890

Karczewski KJ, Francioli LC, Tiao G et al. (2020). The mutational constraint spectrum quantified from variation in 141,456 humans. Nature 581: 434–443. https://doi: 10.1038/s41586-020-2308-7

Supplementary Material 1: Muscle-specific panel (380 genes)

AAAS, AARS1, ABCC9, ABHD12, ABHD5, ACAD9, ACADL, ACADM, ACADS, ACADVL, ACTA1, ACTN2, ADCY6, ADGRG6, ADSS1, AGL, AGRN, AIFM1, ALDOA, ALG14, ALG2, ALG3, AMPD1, ANO5, ARHGEF10, ASAH1, ASCC1, ATL1, ATL3, ATP1A1, ATP1A2, ATP2A1, ATP7A, B3GALNT2, B4GAT1, BAG3, BICD2, BIN1, BSCL2, BVES, CACNA1E, CACNA1S, CAPN3, CASQ1, CAV3, CAVIN1, CCDC78, CCT5, CFAP276, CFL2, CHAT, CHCHD10, CHKB, CHRNA1, CHRNB1, CHRND, CHRNE, CHRNG, CHST14, CLCN1, CNTN1, CNTNAP1, COA7, COL12A1, COL13A1, COL6A1, COL6A2, COL6A3, COLQ, COX6A1, COX6A2, CPT2, CRPPA, CRYAB, CTDP1, DAG1, DCAF8, DCTN1, DCTN2, DES, DGAT2, DHTKD1, DMD, DNA2, DNAJB2, DNAJB5, DNAJB6, DNM2, DNMT1, DOK7, DPAGT1, DPM1, DPM2, DPM3, DRP2, DST, DYNC1H1, DYSF, ECEL1, EGR2, ELP1, EMD, EMILIN1, ENO3, ERBB3, ERGIC1, ETFA, ETFB, ETFDH, EXOSC3, EXOSC8, FBLN5, FBN2, FBXO38, FGD4, FHL1, FIG4, FKBP10, FKBP14, FKRP, FKTN, FLAD1, FLNB, FLNC, FXR1, G6PC1, GAA, GAN, GARS1, GBE1, GDAP1, GFPT1, GJB1, GJB3, GLA, GLDN, GLE1, GMPPB, GNB4, GNE, GOLGA2, GSN, GYG1, GYS1, HACD1, HADH, HADHA, HADHB, HARS1, HEXA, HINT1, HK1, HNRNPDL, HOXD10, HSPB1, HSPB3, HSPB8, IGHMBP2, INF2, INPP5K, ISCU, ITGA7, KARS1, KBTBD13, KCNE3, KCNJ2, KCNJ5, KIF1A, KIF1B, KIF5A, KLHL40, KLHL41, KLHL9, KY, LAMA2, LAMA5, LAMP2, LARGE1, LAS1L, LDB3, LDHA, LGI4, LIMS2, LITAF, LMNA, LMOD3, LPIN1, LRP12, LRP4, LRSAM1, MAP3K20,

MARS1, MATR3, MB, MCM3AP, MED25, MEGF10, MET, MFN2, MICU1, MME, MORC2, MPV17, MPZ, MSTO1, MTM1, MTMR14, MTMR2, MTRFR, MUSK, MYBPC1, MYH14, MYH2, MYH3, MYH7, MYH8, MYL1, MYO9A, MYOD1, MYOF, MYOT, MYPN, NAGLU, NALCN, NDRG1, NEB, NEFH, NEFL, NEK9, NGF, NHERF1, NPL, NTRK1, NUP88, OPA1, ORAI1, PABPN1, PAX7, PDHA1, PDK3, PDXK, PFKM, PGAM2, PGK1, PGM1, PHKA1, PHKB, PHKG2, PIEZO2, PIP5K1C, PLEC, PLEKHG5, PLOD2, PMP2, PMP22, PNPLA2, POGLUT1, POLG, POLG2, POMGNT1, POMGNT2, POMK, POMT1, POMT2, POPDC3, PPP3CA, PRDM12, PREPL, PRKAG2, PRPS1, PRX, PUS1, PYGM, PYROXD1, RAB7A, RAPSN, RBCK1, RBM7, REEP1, RETREG1, RRM2B, RXYLT1, RYR1, RYR3, SBF1, SBF2, SCARF2, SCN10A, SCN11A, SCN4A, SCN9A, SCO2, SCYL2, SELENON, SEPTIN9, SETX, SEPN1, SGCA, SGCB, SGCD, SGCG, SGPL1, SH3TC2, SIGMAR1, SIL1, SLC12A6, SLC16A1, SLC18A3, SLC22A5, SLC25A1, SLC25A20, SLC25A21, SLC25A42, SLC25A46, SLC5A7, SMCHD1, SMPD4, SNAP25, SOD1, SORD, SOX10, SPEG, SPG11, SPTAN1, SPTBN4, SPTLC1, SPTLC2, STAC3, STIM1, SUCLA2, SURF1, SYNE1, SYNE2, SYT2, TAFAZZIN, TCAP, TECPR2, TFG, TIA1, TIMM22, TK2, TMEM43, TMEM65, TNNI2, TNNT1, TNNT3, TNPO3, TOR1A, TOR1AIP1, TPM2, TPM3, TRAPPC11, TRIM2, TRIM32, TRIM54, TRIM63, TRIP4, TRPA1, TRPV4, TTN, TTR, TWNK, TYMP, UBA1, UBA5, UNC50, VAMP1, VAPB, VCP, VIPAS39, VMA21, VPS33B, VRK1, VWA1, WARS1, WNK1, YARS1, YARS2, ZC4H2, ZFHX2

Supplementary Table1: Genetic findings

**Gene**	**Variant**	
CAPN3	NM_000070.3:c.1865_1866delAG p.Glu622Glyfs*9; rs2054085839	Index patient, Father
ACTN2		Index patient, Father
PLEC		Index patient, Father
POLG		Index patient, Mother
RBM20		Index patient, Mother
SGCD		Index patient, Father
SYNE2		Index patient, Mother
OBSCN	NM_001271223.3:c.8724_8725del, p.Gly2909Alafs*2	Index patient, Mother
OBSCN	NM_001271223.3:c.14591C>A, p.Ala4864Asp;	Index patient, Father

RNA isolation, reverse transcription, quantitative PCR

Total RNA was extracted from whole blood and muscle tissue of the index patient and healthy controls (two muscle tissue and nine blood samples) using the Monarch total RNA mini kit

(New England Biolabs, Ipswich, MA, United States). An additional DNase I digestion (Invitrogen, Waltham, MA, United States) was performed to remove traces of genomic DNA. Up to 120 ng each of RNA (derived from blood, and muscle tissue) was transcribed into cDNA using random primers and SuperScript III reverse transcriptase (Invitrogen, Waltham, MA, USA). Prepared cDNA was diluted to the same concentration. The initial input of total RNA was 7 ng per well for quantitative PCR (qPCR).

Quantitative PCR (qPCR) was performed on a CFX384 cycler (Bio-Rad Laboratories, Hercules, USA) using iTaq Universal SYBR Green Supermix (Bio-Rad). Primers used for the detection of *OBSCN* Ex31-34 and Ex35-36, and *EEF2* are listed in Supplementary Table 1. The relative transcript levels of *OBSCN* Ex31-34, and Ex35-36 compared to control samples, were calculated using the ΔΔCT method. *EEF2* was used as reference gene to calculate ΔCT values (CT(*OBSCN*)-CT(*EEF2*)). Obtained data were compared with findings in two muscle samples and nine lymphoblast pellets from healthy controls, respectively. All experiments were performed in technical triplicate. Statistical analysis of ΔCT values was performed by two-tailed unpaired t-test for muscle samples and one-sample t-test for blood samples. Standard deviations and standard errors of the mean were defined.

Supplementary Table 2: Primer sequences for A) *OBSCN* sequencing and B) quantitative RT-PCR

**Primer**	**Sequence 5'- 3'**	**Application**
**A**		*OBSCN* sequencing
OBSCN_Ex33F	GGAATGGTGGACACAGCTCAG	
OBSCN_Ex33R	CGCTGCAAGAGACACACCG	
OBSCN_Ex55F	GCAGCACTGCCTGTGGAG	
OBSCN_Ex55R	GTGGAGCCGTCTCAGTTGG	
**B**		quantitative RT-PCR
OBSCN_cEx31F	CGAGCTGGTCAGTGATGG	
OBSCN_cEx34R	GGACAGCTCACAGGAGAAG	
OBSCN_Ex35F	CCCAGTGACAAGTATGACTTCC	
OBSCN_Ex36R	CACTGTCTTCCCACCGAC	
EEF2_F	CCTTGTGGAGATCCAGTGTCC	
EEF2_R	CTCGTTGACGGGCAGATAGG	

Muscle pathology

A muscle sample was taken and processed according to standard procedures. Enzymatic and immunohistochemical staining was performed at 6 μm thick cryosections. Immunohistochemistry with antibodies against desmin, c5b9, caveolin3, dysferlin, MHCfast and LC3 was performed using a benchmark automatic staining platform (Ventana, Heidelberg, Germany, visualised with Ultra View DAB Detection Kit v1.02.0018, Ventana Medical Systems). For double immunofluorescence staining 8μm thick frozen sections were fixed with cold 100% acetone for 10 minutes, washed, and then blocked with BlockAid (B10710, life Technologies) for one hour at room temperature (RT). Primary antibodies (anti-desmin and anti-obscurin) were diluted with BlockAid, applied to the sections and incubated overnight at 4°C. The next day, secondary antibodies (Alexa Fluor Fluor®568 and Alexa Fluor®488) were applied after further washing steps and incubated for two hours at room temperature (RT). Then, the sections washed and incubated with DAPI-Working Solution (D1306, ThermoFisher Scientific) for 10 minutes. After that, the slides were mounted using Anti-Fade Fluorescence Mounting Medium (104135, Abcam). Sections were analysed with a spinning disk confocal (Yokogawa Benchtop High Content Analysis). For transmission electron microscopy (TEM), small samples were fixed with 6% glutaraldehyde/0.4 M PBS and were processed according to standard protocols. The sections were examined and photographed using a transmission electron Zeiss EM 109 transmission microscope with a 2K-CCD-Camera from TRS.

Supplementary Table 3: Antibodies

**Antibody**	**Species**	**Dilution (Cryosection)**	**Company**
Anti-Human Desmin	Mouse monoclonal	1:1000	DAKO, USA, M0760
Anti-Human C5b-9	Mouse monoclonal	1:50	DAKO, USA, M0777
Purified Mouse Anti-Caveolin 3	Mouse monoclonal	1:500	BD Transduction Laboratories. USA, 610420
Dysferlin	Mouse monoclonal	1:50	Leica, Germany, NCL-Hamlet
LC3	Mouse monoclonal	1:100	nanoTools Germany, 0231-100/LC3-5F10
Anti-MHCfast	Mouse monoclonal	1:100	Leica Biosystems, Clone: WB-MHCF
Anti-Human Desmin	Mouse monoclonal	1:200 (IF)	DAKO, USA, M0760
Anti-Obscurin	Rabbit polyclonal	1:100 (IF)	PA5-54345, Invitrogen
Alexa Fluor®568	Goat anti-rabbit	1:200 (IF)	Abcam
Alexa Fluor®488	Goat anti-mouse	1:200 (IF)	Abcam

Proteomic analysis

Supplementary Table 4: Differentially abundant proteins (DAPs) in the index patient

Upregulated Proteins

Protein Accession number	Gene	Protein	Unique peptides	001-25/Ctrl	P-value
Q13642	*FHL1*	Four and a half LIM domains protein 1	27	65,13	0,00
Q96FJ2	*DYNLL2*	Dynein light chain 2, cytoplasmic	2	17,30	0,00
P50461	*CSRP3*	Cysteine and glycine-rich protein 3	15	16,89	0,00
Q13203	*MYBPH*	Myosin-binding protein H	29	13,11	0,00
Q8IVN3	*MUSTN1*	Musculoskeletal embryonic nuclear protein 1	8	8,26	0,00
A4UGR9	*XIRP2*	Xin actin-binding repeat-containing protein 2	126	8,20	0,05
P48681	*NES*	Nestin	42	7,46	0,00
P01860	*IGHG3*	Immunoglobulin heavy constant gamma 3	5	6,32	0,04
P62841	*RPS15*	40S ribosomal protein S15	2	6,16	0,01
O43598	*DNPH1*	2'-deoxynucleoside 5'-phosphate N-hydrolase 1	2	6,06	0,01
Q86VF7	*NRAP*	Nebulin-related-anchoring protein	114	5,61	0,03
Q13643	*FHL3*	Four and a half LIM domains protein 3	16	5,42	0,03
P09936	*UCHL1*	Ubiquitin carboxyl-terminal hydrolase isozyme L1	11	5,26	0,01
Q13045	*FLII*	Protein flightless-1 homolog	4	5,25	0,03
P35637	*FUS*	RNA-binding protein FUS	2	5,09	0,01
Q9UH99	*SUN2*	SUN domain-containing protein 2	3	5,06	0,00
O75832	*PSMD10*	26S proteasome non-ATPase regulatory subunit 10	2	4,28	0,01
P54105	*CLNS1A*	Methylosome subunit pICln	2	4,09	0,01
Q9Y6G9	*DYNC1LI1*	Cytoplasmic dynein 1 light intermediate chain 1	6	4,08	0,00
Q9Y371	*SH3GLB1*	Endophilin-B1	3	3,92	0,00
Q9UKX2	*MYH2*	Myosin-2	91	3,76	0,01
P62910	*RPL32*	60S ribosomal protein L32	2	3,60	0,04
P09972	*ALDOC*	Fructose-bisphosphate aldolase C	2	3,56	0,02
P17661	*DES*	Desmin	57	3,48	0,00
P62873	*GNB1*	Guanine nucleotide-binding protein G(I)/G(S)/G(T) subunit beta-1	3	3,46	0,03
Q9Y570	*PPME1*	Protein phosphatase methylesterase 1	2	3,38	0,03
P11182	*DBT*	Lipoamide acyltransferase component of branched-chain alpha-keto acid dehydrogenase complex, mitochondrial	3	3,32	0,02

P48444	*ARCN1*	Coatomer subunit delta	2	3,26	0,02
P41567	*EIF1*	Eukaryotic translation initiation factor 1	2	3,18	0,01
O76003	*GLRX3*	Glutaredoxin-3	4	3,15	0,01
Q9UJY1	*HSPB8*	Heat shock protein beta-8	10	2,97	0,00
Q8N3D4	*EHBP1L1*	EH domain-binding protein 1-like protein 1	11	2,87	0,01
P48059	*LIMS1*	LIM and senescent cell antigen-like-containing domain protein 1	3	2,84	0,04
P53999	*SUB1*	Activated RNA polymerase II transcriptional coactivator p15	2	2,77	0,03
Q7Z7K6	*CENPV*	Centromere protein V	3	2,73	0,00
Q96CT7	*CCDC124*	Coiled-coil domain-containing protein 124	4	2,67	0,00
P36959	*GMPR*	GMP reductase 1	5	2,64	0,03
P00441	*SOD1*	Superoxide dismutase [Cu-Zn]	10	2,63	0,01
Q8WX93	*PALLD*	Palladin	13	2,59	0,01
Q702N8	*XIRP1*	Xin actin-binding repeat-containing protein 1	70	2,56	0,02
P10636	*MAPT*	Microtubule-associated protein tau	15	2,53	0,01
Q0ZGT2	*NEXN*	Nexilin	20	2,52	0,01
P52294	*KPNA1*	Importin subunit alpha-5	6	2,50	0,00
Q13557	*CAMK2D*	Calcium/calmodulin-dependent protein kinase type II subunit delta	18	2,44	0,02
O94760	*DDAH1*	N(G),N(G)-dimethylarginine dimethylaminohydrolase 1	10	2,40	0,00
Q9Y3U8	*RPL36*	60S ribosomal protein L36	2	2,40	0,02
Q04917	*YWHAH*	14-3-3 protein eta	8	2,37	0,04
Q92614	*MYO18A*	Unconventional myosin-XVIIIa	33	2,35	0,02
Q93009	*USP7*	Ubiquitin carboxyl-terminal hydrolase 7	2	2,35	0,01
Q8NDY3	*ADPRHL1*	Inactive ADP-ribosyltransferase ARH2	14	2,31	0,00
Q96BM9	*ARL8A*	ADP-ribosylation factor-like protein 8A	4	2,31	0,03
Q00765	*REEP5*	Receptor expression-enhancing protein 5	8	2,29	0,00
O00233	*PSMD9*	26S proteasome non-ATPase regulatory subunit 9	3	2,29	0,00
P13535	*MYH8*	Myosin-8	51	2,28	0,02
Q9HB71	*CACYBP*	Calcyclin-binding protein	3	2,27	0,04
P62495	*ETF1*	Eukaryotic peptide chain release factor subunit 1	3	2,27	0,04
Q9BXI3	*NT5C1A*	Cytosolic 5'-nucleotidase 1A	17	2,27	0,00
P62070	*RRAS2*	Ras-related protein R-Ras2	4	2,23	0,02
Q96QR8	*PURB*	Transcriptional activator protein Pur-beta	5	2,22	0,00

O60841	*EIF5B*	Eukaryotic translation initiation factor 5B	3	2,22	0,00
Q13347	*EIF3I*	Eukaryotic translation initiation factor 3 subunit I	5	2,21	0,01
Q2TBA0	*KLHL40*	Kelch-like protein 40	26	2,19	0,00
Q8WVM8	*SCFD1*	Sec1 family domain-containing protein 1	5	2,17	0,00
O95817	*BAG3*	BAG family molecular chaperone regulator 3	30	2,15	0,00
P08708	*RPS17*	40S ribosomal protein S17	5	2,15	0,03
P16152	*CBR1*	Carbonyl reductase [NADPH] 1	17	2,14	0,03
P62633	*CNBP*	CCHC-type zinc finger nucleic acid binding protein	6	2,14	0,05
Q15056	*EIF4H*	Eukaryotic translation initiation factor 4H	7	2,12	0,00
P23327	*HRC*	Sarcoplasmic reticulum histidine-rich calcium-binding protein	8	2,12	0,04
P42330	*AKR1C3*	Aldo-keto reductase family 1 member C3	9	2,11	0,00
O60662	*KLHL41*	Kelch-like protein 41	51	2,11	0,02

Downregulated Proteins

Protein Accessions number	Gene	Protein	Unique peptides	001-25/Ctrl	P-value
Q02252	*ALDH6A1*	Methylmalonate-semialdehyde dehydrogenase [acylating], mitochondrial	7	0,50	0,01
O00264	*PGRMC1*	Membrane-associated progesterone receptor component 1	2	0,50	0,04
Q13404	*UBE2V1*	Ubiquitin-conjugating enzyme E2 variant 1	8	0,49	0,03
Q8IW45	*NAXD*	ATP-dependent (S)-NAD(P)H-hydrate dehydratase	7	0,49	0,00
Q9Y235	*APOBEC2*	C->U-editing enzyme APOBEC-2	13	0,48	0,00
Q9UHY7	*ENOPH1*	Enolase-phosphatase E1	4	0,48	0,04
A5D6W6	*FITM1*	Fat storage-inducing transmembrane protein 1	4	0,47	0,01
Q01484	*ANK2*	Ankyrin-2	3	0,47	0,05
P30043	*BLVRB*	Flavin reductase (NADPH)	12	0,47	0,03
Q6JBY9	*RCSD1*	CapZ-interacting protein	2	0,47	0,02
Q8N335	*GPD1L*	Glycerol-3-phosphate dehydrogenase 1-like protein	17	0,47	0,02
Q8N8N7	*PTGR2*	Prostaglandin reductase 2	8	0,46	0,02
O75390	*CS*	Citrate synthase, mitochondrial	21	0,46	0,05
Q01469	*FABP5*	Fatty acid-binding protein 5	7	0,46	0,01
P40939	*HADHA*	Trifunctional enzyme subunit alpha, mitochondrial	50	0,46	0,05
P12429	*ANXA3*	Annexin A3	5	0,46	0,00
P08134	*RHOC*	Rho-related GTP-binding protein RhoC	7	0,46	0,03
Q13228	*SELENBP1*	Methanethiol oxidase	21	0,45	0,01
Q96Q06	*PLIN4*	Perilipin-4	47	0,45	0,01
P60660	*MYL6*	Myosin light polypeptide 6	5	0,45	0,00
O75521	*ECI2*	Enoyl-CoA delta isomerase 2	7	0,45	0,00
P61586	*RHOA*	Transforming protein RhoA	2	0,45	0,01
P61088	*UBE2N*	Ubiquitin-conjugating enzyme E2 N	10	0,44	0,00

Q8NCW5	*NAXE*	NAD(P)H-hydrate epimerase	10	0,43	0,04
P62820	*RAB1A*	Ras-related protein Rab-1A	2	0,42	0,05
P49419	*ALDH7A1*	Alpha-aminoadipic semialdehyde dehydrogenase	7	0,42	0,04
P11177	*PDHB*	Pyruvate dehydrogenase E1 component subunit beta, mitochondrial	17	0,41	0,04
Q9Y6B6	*SAR1B*	GTP-binding protein SAR1b	4	0,41	0,03
Q00796	*SORD*	Sorbitol dehydrogenase	3	0,41	0,01
O43169	*CYB5B*	Cytochrome b5 type B	3	0,41	0,00
Q9Y376	*CAB39*	Calcium-binding protein 39	9	0,41	0,02
P11310	*ACADM*	Medium-chain specific acyl-CoA dehydrogenase, mitochondrial	20	0,39	0,02
P08559	*PDHA1*	Pyruvate dehydrogenase E1 component subunit alpha, somatic form, mitochondrial	24	0,37	0,05
Q8TCA0	*LRRC20*	Leucine-rich repeat-containing protein 20	8	0,37	0,01
P30044	*PRDX5*	Peroxiredoxin-5, mitochondrial	11	0,36	0,01
O60256	*PRPSAP2*	Phosphoribosyl pyrophosphate synthase-associated protein 2	3	0,36	0,00
Q16762	*TST*	Thiosulfate sulfurtransferase	5	0,36	0,04
P49748	*ACADVL*	Very long-chain specific acyl-CoA dehydrogenase, mitochondrial	36	0,36	0,05
P13716	*ALAD*	Delta-aminolevulinic acid dehydratase	6	0,35	0,03
P11233	*RALA*	Ras-related protein Ral-A	2	0,34	0,01
P14649	*MYL6B*	Myosin light chain 6B	24	0,33	0,02
P42126	*ECI1*	Enoyl-CoA delta isomerase 1, mitochondrial	8	0,33	0,01
P21980	*TGM2*	Protein-glutamine gamma-glutamyltransferase 2	6	0,33	0,05
O94811	*TPPP*	Tubulin polymerisation-promoting protein	2	0,33	0,01
P35754	*GLRX*	Glutaredoxin-1	6	0,32	0,03
P55822	*SH3BGR*	SH3 domain-binding glutamic acid-rich protein	5	0,32	0,00
P27338	*MAOB*	Amine oxidase [flavin-containing] B	10	0,32	0,01

P16615	*ATP2A2*	Sarcoplasmic/endoplasmic reticulum calcium ATPase 2	35	0,32	0,05
Q58FF8	*HSP90AB2P*	Putative heat shock protein HSP 90-beta 2	2	0,31	0,05
O75208	*COQ9*	Ubiquinone biosynthesis protein COQ9, mitochondrial	9	0,30	0,03
P61086	*UBE2K*	Ubiquitin-conjugating enzyme E2 K	3	0,30	0,02
O75531	*BANF1*	Barrier-to-autointegration factor	5	0,30	0,01
P63208	*SKP1*	S-phase kinase-associated protein 1	2	0,28	0,05
Q5XKP0	*MICOS13*	MICOS complex subunit MIC13	5	0,27	0,03
Q9H6F2	*TMEM38A*	Trimeric intracellular cation channel type A	4	0,27	0,03
Q9BXS5	*AP1M1*	AP-1 complex subunit mu-1	3	0,26	0,00
P13533	*MYH6*	Myosin-6	8	0,26	0,03
P07108	*DBI*	Acyl-CoA-binding protein	6	0,25	0,01
Q52LJ0	*FAM98B*	Protein FAM98B	4	0,25	0,00
P24310	*COX7A1*	Cytochrome c oxidase subunit 7A1, mitochondrial	3	0,22	0,01
P02042	*HBD*	Hemoglobin subunit delta	10	0,21	0,05
Q9H0N5	*PCBD2*	Pterin-4-alpha-carbinolamine dehydratase 2	3	0,21	0,00
P13645	*KRT10*	Keratin, type I cytoskeletal 10	29	0,21	0,04
Q86Y39	*NDUFA11*	NADH dehydrogenase [ubiquinone] 1 alpha subcomplex subunit 11	5	0,20	0,03
P16157	*ANK1*	Ankyrin-1	10	0,18	0,05
P05387	*RPLP2*	60S acidic ribosomal protein P2	3	0,17	0,00
P02730	*SLC4A1*	Band 3 anion transport protein	17	0,15	0,04
P02753	*RBP4*	Retinol-binding protein 4	2	0,15	0,02
O60936	*NOL3*	Nucleolar protein 3	2	0,04	0,01
P00491	*PNP*	Purine nucleoside phosphorylase	2	0,04	0,02

Supplementary Figure 1:

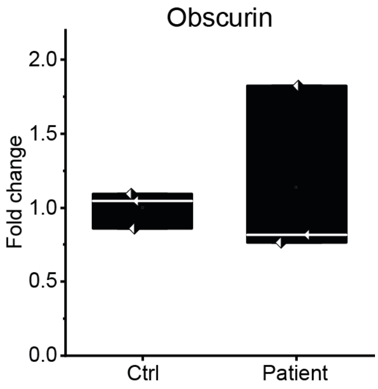

Suppl. Fig. 1: Box-plot of protein abundance of obscurin in the patient compared to three controls (Crtl).

Supplementary Figure 2:

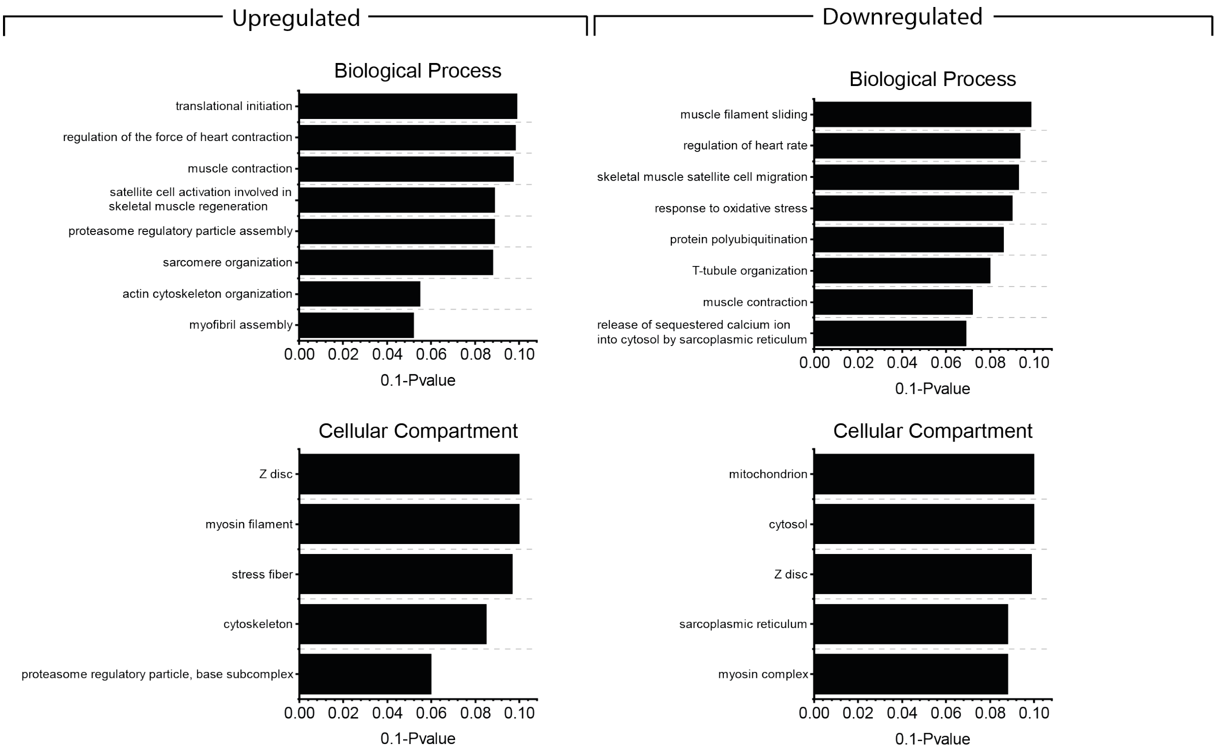

Suppl. Fig. 2: Deregulated GO terms in the patient compared to three controls.

The Graphical abstract was created in BioRender. Schänzer, A. (2025) https://BioRender.com/gcj9our
